# Biophysical characterization of the b-HLH-LZ of ΔMax, an alternatively spliced isoform of Max found in tumor cells: Towards the validation of a tumor suppressor role for the Max homodimers

**DOI:** 10.1371/journal.pone.0174413

**Published:** 2017-03-28

**Authors:** Loïka Maltais, Martin Montagne, Mikaël Bédard, Cynthia Tremblay, Laura Soucek, Pierre Lavigne

**Affiliations:** 1 Département de Biochimie, Faculté de Médecine et des Sciences de la Santé, Institut de Pharmacologie de Sherbrooke, Université de Sherbrooke, Sherbrooke, Québec, Canada; 2 PROTÉO; Regroupement Stratégique sur la Fonction, la Structure et l'Ingénierie des Protéines, Université Laval, Québec, Canada; 3 Vall d’Hebron Institute of Oncology (VHIO), Hospital Vall d’Hebron, Barcelona, Spain; 4 Institució Catalana de Recerca i Estudis Avançats (ICREA), Barcelona, Spain; 5 Department of Biochemistry and Molecular Biology, Universitat Autònoma de Barcelona, Bellaterra, Spain; Saint Louis University, UNITED STATES

## Abstract

It is classically recognized that the physiological and oncogenic functions of Myc proteins depend on specific DNA binding enabled by the dimerization of its C-terminal basic-region-Helix-Loop-Helix-Leucine Zipper (b-HLH-LZ) domain with that of Max. However, a new paradigm is emerging, where the binding of the c-Myc/Max heterodimer to non-specific sequences in enhancers and promoters drives the transcription of genes involved in diverse oncogenic programs. Importantly, Max can form a stable homodimer even in the presence of c-Myc and bind DNA (specific and non-specific) with comparable affinity to the c-Myc/Max heterodimer. Intriguingly, alterations in the Max gene by germline and somatic mutations or changes in the gene product by alternative splicing (e.g. ΔMax) were recently associated with pheochromocytoma and glioblastoma, respectively. This has led to the proposition that Max is, by itself, a tumor suppressor. However, the actual mechanism through which it exerts such an activity remains to be elucidated. Here, we show that contrary to the WT motif, the b-HLH-LZ of ΔMax does not homodimerize in the absence of DNA. In addition, although ΔMax can still bind the E-box sequence as a homodimer, it cannot bind non-specific DNA in that form, while it can heterodimerize with c-Myc and bind E-box and non-specific DNA as a heterodimer with high affinity. Taken together, our results suggest that the WT Max homodimer is important for attenuating the binding of c-Myc to specific and non-specific DNA, whereas ΔMax is unable to do so. Conversely, the splicing of Max into ΔMax could provoke an increase in overall chromatin bound c-Myc. According to the new emerging paradigm, the splicing event and the stark reduction in homodimer stability and DNA binding should promote tumorigenesis impairing the tumor suppressor activity of the WT homodimer of Max.

## Introduction

Myc proteins (N-, L- and c-Myc) are basic region-Helix-Loop-Helix-Leucine Zipper (b-HLH-LZ) transcriptional regulators with a broad spectrum of physiological and oncogenic target genes [1–3]. While essential for normal development, cell growth and proliferation as well as apoptosis, they play a major role in cancer onset and progression when deregulated [4]. Physiological and oncogenic functions of Myc proteins are facilitated by the specific heterodimerization with Max through their b-HLH-LZ domains [1,5]. Sustained expression and persistent levels of Myc proteins caused by mutations in other oncogenes such as *EGFR* and *RAS* can amplify the transcription of oncogenic genes or programs and lead to the addiction of tumor cells to such programs [6]. This mechanism of addiction is now strongly supported by studies and data that demonstrate the invasion of transcriptionally active chromatin (enhancers and promoters)—often devoid of E-box sequences—by persistent and high levels of Myc [7,8]. As described below, this spill-over to sites where no E-boxes are found can be explained by the fact that b-HLH-LZ transcription factors can bind to E-box and non-E-box DNA sequences with high and almost comparable affinities [9].

Recently, an alternatively spliced isoform of Max (ΔMax) identified in numerous cancer cells [10] has been reported to increase Myc transcriptional activities in glioblastoma multiforme (GBM) cell lines [11]. More precisely, Babic et al. showed that the pre-messenger RNA of WT Max is spliced by hnRNPA1, whose transcription is activated by high and persistent levels of c-Myc in GBM primary cell cultures and immortalized cell lines. Indeed, high levels of c-Myc correlate with a lower ratio of WT/ΔMax mRNA and with the expression of metabolic genes, which drive tumor cell proliferation. Moreover, lower ratios of WT/ΔMax mRNA correlate with the aggressiveness of GBM lesions [11]. Intriguingly, whereas the ectopic overexpression of ΔMax potentiates Myc transcriptional activities and proliferation in GBM cell lines, the overexpression of WT Max was shown to have the opposite effect. Although similar antagonistic effects of the ectopic expression of Max have been reported [12–14], the mechanism underlying the stark differences between ΔMax and Max on Myc transcriptional activities is yet to be explained.

ΔMax is the result of the inclusion of exon 5, which leads to a shorter C-terminus and a change in primary structure in the last heptad of its LZ. Here, we show for the first time to our knowledge that this modification of the primary structure of the LZ leads to a b-HLH-LZ domain that is incapable of forming a stable homodimer. However, the b-HLH-LZ domain of ΔMax can still form a heterodimer with the b-HLH-LZ of c-Myc. Moreover, whereas the b-HLH-LZ of ΔMax can bind E-box sequences, it can no longer bind non-specific DNA like the WT counterpart. Furthermore, the b-HLH-LZ of ΔMax can bind both the E-box and non-specific DNA as a heterodimer with the b-HLH-LZ of c-Myc. Altogether, our data indicate that, contrary to the WT Max homodimer, ΔMax cannot attenuate the binding of c-Myc to E-box sequences and non-specific DNA and would, instead, promote the binding of deregulated c-Myc to E-box and non-specific sites. Our data also advocate the notion that the ability of WT Max to form a homodimer and bind specific and non-specific DNA bestows it with a tumor suppressor role.

## Results

### Modeling of the homodimeric LZ of ΔMax and heterodimeric c-Myc/ΔMax LZ

**Fig 1:**
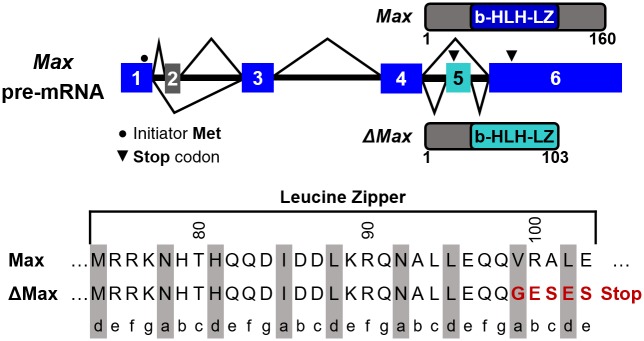
Alternative splicing of WT Max leads to the expression of ΔMax. ΔMax is generated by exon 5 inclusion. This leads to a shorter LZ and a change in the primary structure of the last heptad.

**Fig 2:**
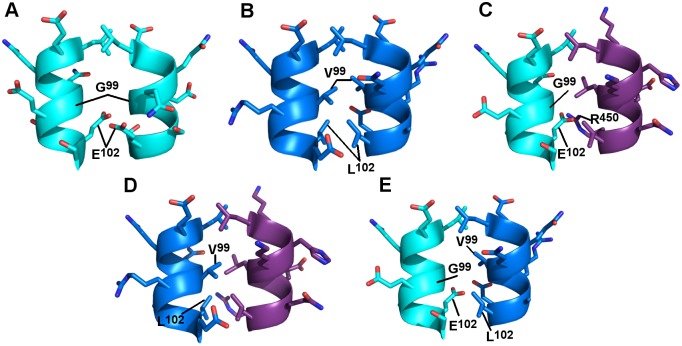
Models of the last heptad of ΔMax and WT Max. Models of the last heptad of the LZ of ΔMax (**A**) and WT Max (**B**) in a homodimeric state. Models of the last heptad of the LZs of ΔMax/c-Myc (**C**), WT Max/c-Myc (**D**) and ΔMax/WT Max (**E**) in a heterodimeric state. Although various structures of the Max homodimeric and c-Myc/Max b-HLH-LZ have been reported [16,17], the last heptad is incomplete or ill-defined in all of them. They were therefore remodeled along with the LZ of ΔMax using the program Pymol (http://www.pymol.org) and the backbone of the structure of the heterodimeric c-Myc/Max (1A93) as a template [18].

The ΔMax isoform, identified 20 years ago, results from the inclusion of exon 5 of the Max gene by alternative splicing [10,15]. This leads to a shorter protein and a significant modification in the primary structure of the last heptad of the LZ (Fig 1). The change in primary structure includes the replacement of the hydrophobic residues Val and Leu at positions **a** and **d** of the final heptad repeat by a Gly and a Glu, respectively (Fig 2A and 2B). Such a change of interfacial residues can be anticipated to destabilize the homodimeric LZ by the reduction in hydrophobic interactions and the introduction of electrostatic repulsions.

The thermodynamical destabilization of homodimeric species is an integral part of the mechanism of specific heterodimerization, because it favors heteromeric encounters [18–23]. In addition to this macroscopic and probabilistic effect, relief of the destabilizing interactions brought about by charged residues at the homodimer interface by favorable interaction at the interface of the heterodimer will further ensure that the latter is thermodynamically stable and hence almost exclusively formed. As discussed extensively elsewhere, the molecular recognition between WT c-Myc and WT Max relies on the relief of strong (Myc) and moderate (Max) destabilizing electrostatic interactions of the respective homodimeric LZs to the profit of a more stable interfacial salt bridge between conserved acidic side-chain on c-Myc and the His side-chain on Max at position **d** [18,19]. Additional electrostatic interactions have been proposed by others to contribute to the heterodimerization process [17].

Inspection of the model of the heterodimeric LZ between ΔMax and c-Myc indicates that the formation of the heterodimer should be more favorable. Indeed, the repulsion between the two Glu residues at position **d** are no longer present and a potential charged H-bond could form between the side-chains of the Glu on ΔMax and the Arg at position **e** on the c-Myc LZ. Moreover, the Leu at position **d** on the LZ of c-Myc can engage hydrophobic interactions with the Cβ and Cγ of the Gln side-chain at position **g** on the LZ of ΔMax (Fig 2C). However, it is expected that the thermodynamical stability of the ΔMax/c-Myc dimer will be less than the WT counterpart, due to a lesser content in interfacial hydrophobic interactions (Fig 2D). Accordingly, a similar situation can be observed in the case of the heterodimeric LZ of ΔMax and WT Max (Fig 2E).

### The b-HLH-LZ of ΔMax* does not fold into a stable homodimer in absence of DNA

**Fig 3:**
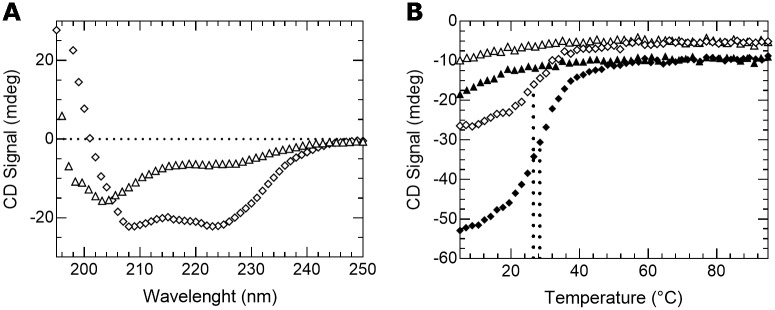
ΔMax* does not form a stable homodimer. (**A**) Far-UV CD spectra of ΔMax* (open triangles) and Max* (open diamonds). (**B**) Thermal denaturations of ΔMax* (triangles) and Max* (diamonds) recorded at 12 μM (open) and 24 μM (black) by monitoring the CD signal at 222 nm. The melting temperatures (T°) of Max* are indicated by the dotted lines.

We present on Fig 3A the far-UV CD (circular dichroism) spectra of the b-HLH-LZs of Max (Max*) and ΔMax (ΔMax*) at 20°C, 12 μM and neutral pH in the absence of DNA. As described in detail before [24,25], under such conditions, Max* forms a homodimer with apparent K_D_s (dissociation constant or the reciprocal of the dimerization constant (1/K_dimer_)) of 1·10^−6^. and 8·10^−4^ at 20°C and 37°C, respectively. Hence, the resulting spectrum is typical of a α-helical dimeric protein with minima at 208 and 222 nm. Conversely, as expected from the change in primary structure in its LZ (Fig 2), the far-UV CD spectrum of ΔMax* with a deep minimum around 205 nm and a shallow signal around 222 nm is more typical of a mixture between residual α-helices and a mainly unfolded protein indicative of a very unstable homodimer.

To further characterize the lack of an appreciable population of ΔMax* homodimers, we have measured the temperature dependence of the CD signal at 222 nm (Fig 3B). Whereas the temperature dependence of the CD signal of Max* displays a cooperative transition of its helical content from its folded and dimeric state to its monomeric and unfolded state, such a cooperative transition is not observed in the case of ΔMax*. This means that the amount of dimeric ΔMax* is insignificant and that its K_D_ in absence of DNA is higher than the 10^−6^ range. To further prove the difference between the K_D_ of ΔMax* and Max*, we have recorded the thermal denaturation at twice the concentration. For a dimer with a K_D_ in the 10^−6^ range like Max*, it is expected that the population of dimer (and apparent T°) will increase, whereas for a dimer with a higher K_D_ like ΔMax*, such a behavior is not expected. Accordingly, as shown in Fig 3B, the T° of Max* is indeed increased from 26,4°C to 28,3°C whereas that of ΔMax* is not. Hence, the data presented in this section clearly demonstrate that the changes in primary structure in the last heptad of the b-HLH-LZ of ΔMax destabilizes its homodimeric form and promotes the population of its monomeric and unfolded state.

### Splicing of Max into ΔMax is predicted to decrease the population of WT Max homodimer in favor of the c-Myc/ΔMax heterodimers

As modeled in Fig 2, it is expected that the destabilizing interactions present at the interface of the homodimeric ΔMax* LZ would instead be relieved at the interface of the heterodimer with c-Myc and allow for molecular recognition. In order to test this hypothesis, we have studied the heterodimerization between c-Myc*, ΔMax* and Max* by CD. An excess of ΔMax* (12 *vs*. 8 μM) was used to promote heteromeric interactions and emphasize the difference with Max*, which can easily homodimerize and limit the amount of heterodimerization.

**Fig 4:**
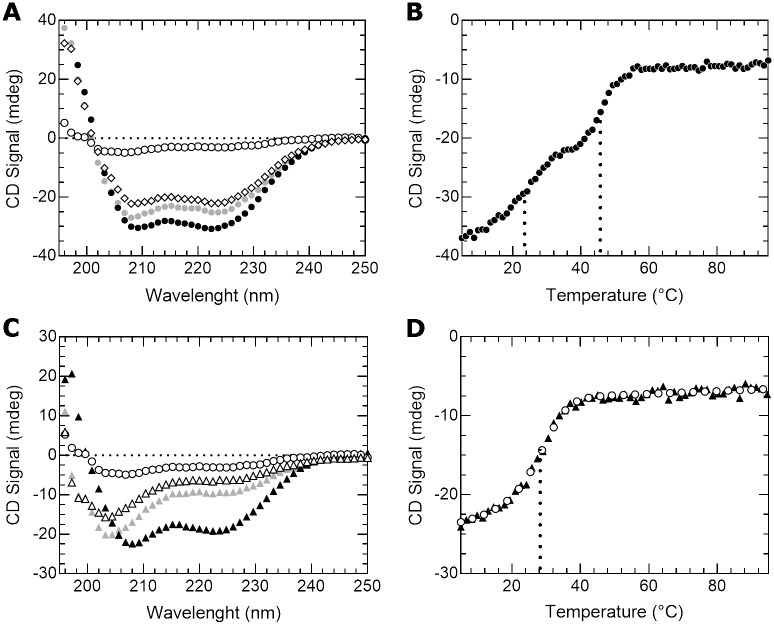
In solution with c-Myc*, Max* forms both homo- and heterodimers while ΔMax* exclusively heterodimerizes. (**A**) Far-UV CD spectra of c-Myc* (open circles), Max* (open diamonds), c-Myc*/Max* (black circles) and the arithmetic sum of the individual spectra (gray circles). (**B**) Thermal denaturation of c-Myc*/Max* (black circles) recorded by monitoring the CD signal at 222 nm. The melting temperatures (T°) of the complexes of the mixture are indicated by the dotted lines. (**C**) Far-UV CD spectra of c-Myc* (open circles), ΔMax* (open triangles), c-Myc*/ΔMax* (black triangles) and the arithmetic sum of the individual spectra (gray triangles). (**D**) Thermal denaturation of c-Myc*/ΔMax* (black triangles) recorded by monitoring the CD signal at 222 nm and simulation of the thermal denaturation of an 8 μM dimer into two unfolded monomers (open circles). The melting temperature (T°) is indicated by the dotted line.

First, we present in Fig 4 the case of Max* and c-Myc*. Helical structure formation and heterodimerization is evident from the difference between the CD signal of the mixture and the arithmetic sum of the individual spectra (Fig 4A). As reported in more detail elsewhere for the equimolar case [26], the thermal denaturation profile obtained (Fig 4B) clearly shows the coexistence of the homodimeric Max* (apparent T° = 23,4°C) and the c-Myc*/Max* heterodimer (apparent T° = 45,7°C). This result underscores the fact that Max* can partition between its homodimeric and heterodimeric states. Indeed, compared to the T° of 26,4°C of pure Max* at 12 μM, the apparent T° of Max* at the same concentration but in the presence of c-Myc* is lowered. This is explained by the fact that the effective concentration available for homodimerization and the equilibrium population of the homodimer are reduced due to its partition into the heterodimer.

The situation is quite different for the ΔMax*/c-Myc* system. Indeed, in the presence of each other, ΔMax* and c-Myc* give rise to the formation of a significant content of α-helical structure, as judged by the difference between the sum of the individual spectra and that of the mixture, which depict much lower and diagnostic CD signal minima at 208 and 222 nm (Fig 4C). This result indicates that a stable heterodimeric species forms between c-Myc* and ΔMax*. To further document this assertion, we performed the thermal denaturation of the mixture (Fig 4D). In accordance, a single cooperative transition is present, suggesting the existence of a stable heterodimer.

In order to determine the apparent K_D_ of the ΔMax*/c-Myc* heterodimer, we have simulated the thermal denaturation curve assuming the unfolding of a dimer with a concentration of 8 μM into two unfolded monomers (Fig 4D) and using the formalism described elsewhere [25]. The detailed results of the simulation are presented in S1 Text. The apparent 1/K_Dimer_ (20°C) and (37°C) determined were 4.9·10^−7^ and 1.6·10^−4^, respectively. These values are larger than those of the WT heterodimer (5.1·10^-7^ (20°C) and 3.5·10^−6^ (37°C)) [23] and demonstrate that, although the ΔMax*/c-Myc* heterodimer can form, it is a less thermodynamically stable heterodimer than WT Max*/c-Myc*. The thermodynamical characterization of the heterodimerization between the WT c-Myc and Max b-HLH-LZs using fluorescence anisotropy and EMSA has also been reported [27–29]. Our values for the the 1/K_Dimer_ for the heterodimer are in complete agreement with those determined by Banerjee et al. [28]. However, and as discussed elsewhere, those obtained by EMSA are significantly higher and probably reflects the fact that those measurements were not made in solution but rather in gels [27]. Overall, our data demonstrate that since ΔMax* does not readily form a homodimer, an increase in the splicing of Max will lead to a decrease in the population of WT Max homodimer in favor of the c-Myc/ΔMax heterodimer.

### The b-HLH-LZ of ΔMax has a significant apparent affinity for the E-box but does not bind non-specific DNA

In order to assess the impact of the primary structure change in the LZ of Max on its DNA binding affinity, we have determined the relative and apparent binding affinities of Max* and ΔMax* for the canonical E-box sequence and non-specific DNA using fluorescence anisotropy. To do so, one has to take into account the dimerization and DNA binding reactions. Indeed, as described in the methods section, the equilibrium constant for the formation of the DNA complex from free b-HLH-LZ (K_Complex_) is the product of two binding constants (K_Complex_ = K_Dimer_·K_Binding_), *i*.*e*. the product of the equilibrium dimerization constant of the b-HLH-LZ in the presence of or on DNA (K_Dimer_) and the equilibrium binding constant of the dimeric b-HLH-LZ to DNA (K_Binding_). As discussed elsewhere [30], whereas the binding of monomer species can occur at room temperature and constitute a kinetic and transient intermediate state *en route* to the formation of the final and most stable dimeric state at equilibrium, it is negligible at 37°C [22].

**Fig 5:**
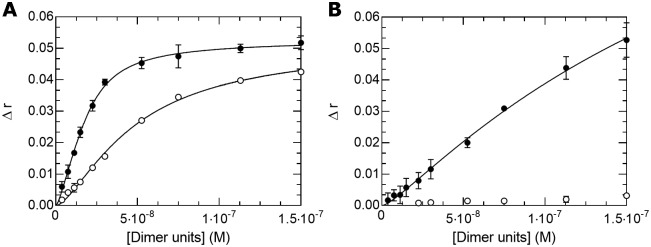
Max* and ΔMax* bind E-box sequences with high affinity. (**A**) Titration of fluorescently labeled E-box (black circles) and non-specific (open circles) sequences with Max*. (**B**) Titration of fluorescently labeled E-box (black circles) and non-specific (open circles) sequences with ΔMax*.

In Fig 5, we present the results of the titration (at 37°C) of fluorescently labeled DNA probes containing a canonical E-box sequence and a non-specific sequence of the same length with Max* and ΔMax*. As discussed above, the population of complex (and the fluorescence anisotropy) as function of the total concentration depends on two equilibrium constants. This represents too many degrees of freedom to obtain meaningful simulations. In this context, we have used a grid search approach to estimate the apparent 1/K_Dimer_ and 1/K_Binding_. More precisely, 1/K_Binding_ values were systematically varied within a range of realistic values and the 1/K_Dimer_ values were obtained using the function described in material and methods by fitting the experimental curves using a least squares method. The combination of constants that best described the titrations was taken to be the one giving a minimum in the statistical error (χ^2^) of the fit (S1 Fig). All the 1/K_Binding_ and 1/K_Dimer_ values obtained are listed in Table 1.

**Table 1:** Results of the analysis of the titration of the E-box and non-specific DNA with the b-HLH-LZs of Max, ΔMax and c-Myc.

	Max*		ΔMax*		ΔMax*/Max*		ΔMax*/c-Myc*		c-Myc*	
	E-box	NS	E-box	NS	E-box	NS	E-box	NS	E-box	NS
**1/K_Binding_ (10^−9^)**	4.8	23	80	N/D	4	40	10	11	70	280
**1/K_Dimer_ (10^−9^)**	8.6 ± 1.2	55 ± 7	220 ± 20	N/D	120 ± 10	460 ± 34	17 ± 2	38 ± 5	0.48 ± 0.5	8.5 ± 3
**Δr max**	0.053 ± 0.001	0.053 ± 0.001	0.116 ± 0.003	N/D	0.063 ± 0.001	0.058 ± 0.001	0.045 ± 0.001	0.048 ± 0.001	0.058 ± 0.001	0.110 ± 0.002

NS, Non-specific DNA; Δr max, Anisotropy ([Dimer]→∞).

We have determined that Max* binds the E-box sequence and the non-specific sequence with apparent 1/K_Binding_ (37°C) of 4.8·10^−9^ and 2.3·10^−8^, respectively, with corresponding 1/K_Dimer_ of 8.6·10^−9^ and 5.5·10^−8^. Also using fluorescence anisotropy, Hu et al. have determined a 1/K_Binding_(21°C) of 20·10^−9^ for the binding of the Max* homodimer to the canonical E-box in a probe of different length and with different flanking sequences [27]. They also reported studies on non-specific sequences. However, those “non-specific” sequences are variations of the CANNTG while we used a scrambled sequence of the same composition of the CACGTG. Although no values were reported for 1/K_Binding_(37°C), it is expected that it would be slightly higher. The temperature difference and the fact that different DNA probes were used makes it difficult to compare both studies in detail. However, both studies agree on the magnitude of the affinity of the Max* homodimer for the canonical E-box. Park et al. have also reported a 1/K_Binding_ that is two order of magnitude higher using EMSA [29].

The apparent 1/K_Dimer_ values determined for Max* and ΔMax* commensurate with the DNA binding affinity indicating a clear linkage between the two processes. However, it is important to point out the important decrease in 1/K_Dimer_ (increase in the stability of the homodimer) in the presence of DNA compared to the scenario where DNA is absent. A similar effect has been observed with poly-anions, where the 1/K_Dimer_ of the b-HLH-LZ of Max was also observed to significantly decrease [28]. This underscores the importance of DNA and the nuclear environment in the efficient dimerization of transcription factors and stresses the fact that the dimerization constant in isolation incompletely describes the probability of occupancy at specific sites. In S2 Fig, we alternately analyzed the formation of the complexes between Max* and the two DNA sequences by CD and demonstrate that the thermodynamical stability (as estimated by the apparent T°) of the complex between Max* and the E-box (T° = 56,1°C) sequence is larger than with the non-specific sequence (T° = 43,7°C). However, whereas ΔMax* binds to the E-box sequence with a sizable affinity with respective apparent 1/K_Binding_ and 1/K_Dimer_ of 8.0·10^−8^ and 2.2·10^−7^, it shows no substantial affinity for the non-specific sequence (Fig 5B). We also show in S2 Fig that the complex between ΔMax* and the E-box is still formed at 37°C (T° = 49,3°C), but that the complex between ΔMax* and the non-specific sequences is only present or stable at room temperature (T° = 26,1°C).

### The b-HLH-LZ of ΔMax binds DNA with the b-HLH-LZ of WT Max with a weaker affinity than the WT homodimer

**Fig 6:**
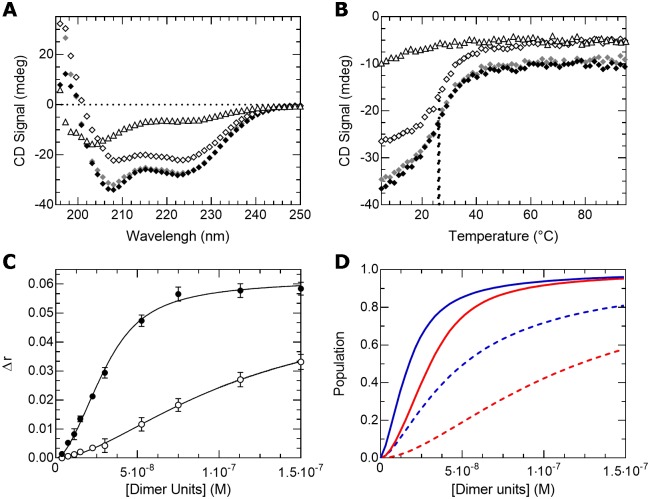
ΔMax* binds E-box and non-specific DNA sequences as a heterodimer with Max*. (**A**) Far-UV CD spectra of ΔMax* (open triangles), Max* (open diamonds), ΔMax*/Max* (black diamonds) and the arithmetic sum of the individual spectra (gray diamonds). (**B**) Thermal denaturations of ΔMax* (open triangles), Max* (open diamonds) and ΔMax*/Max* (black diamonds) recorded by monitoring the CD signal at 222 nm and the arithmetic sum of the individual denaturations (gray diamonds). The melting temperatures (T°) are indicated by the dotted lines. (**C**) Titration of fluorescently labeled E-box (black circles) and non-specific (open circles) sequences with ΔMax*/Max*. (**D**) Populations of Max*/Max* (blue) and ΔMax*/Max* (red) with the E-box (full line) and the non-specific (dotted line) sequences as a function of the total dimeric concentrations.

Because of its inability to homodimerize, an increase in the population of monomeric ΔMax* upon splicing is expected to promote the formation of heteromeric interactions. Hence, we assessed the extent of heterodimerization between ΔMax* and Max*. We present in Fig 6 the Far-UV CD spectra and thermal denaturation of ΔMax* and Max* at 12 μM each, the arithmetic sums, as well as the resulting spectra and denaturation for the mixtures with a total protein concentration of 24 μM. As shown, no difference between the arithmetic sums and the experimental curves are observed, indicating the lack of formation of favorable and stable heterodimeric species in the absence of DNA. As discussed above, this was expected from the modeling of the heterodimeric LZ (Fig 2).

In order to verify if the presence of DNA could stabilize a transient population of heterodimer, we have titrated the E-box and the non-specific sequence with a 1:1 mixture of ΔMax* and Max* (Fig 6C). As shown, both titration curves are well fitted suggesting the binding of a single (and heterodimeric) species in both cases. This is further confirmed by EMSA where a single complex of intermediate apparent molecular weight is detected (between the ΔMax*/ΔMax* and Max*/Max* complexes with the E-box) and by CD, where a single transition is observed (S2 Fig). Compared to the Max* homodimer, we find that the 1/K_Binding_ and 1/K_Dimer_ are larger for both the E-box and non-specific sequences. This indicates that while the ΔMax*/Max* heterodimer can bind the E-box and non-specific sequences, smaller populations of both complexes are formed at equal dimeric concentrations (Fig 6D).

### The c-Myc/ΔMax heterodimeric b-HLH-LZ binds the E-box and non-specific sequence with similar apparent affinities

**Fig 7:**
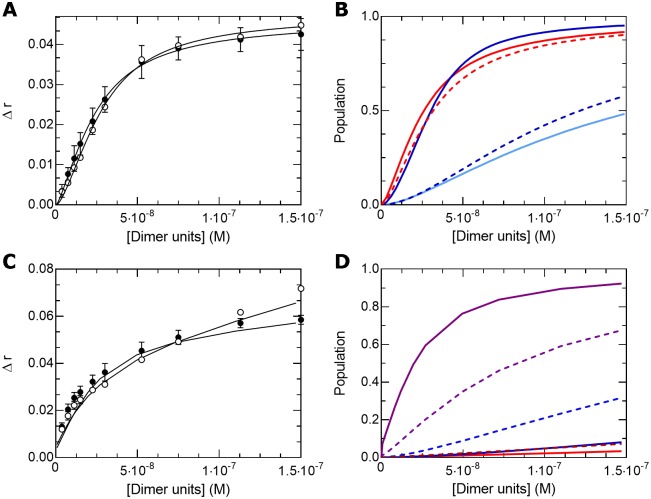
The c-Myc*/ΔMax* heterodimer binds E-Box and non-specific DNA sequences with similar apparent affinities. (**A**) Titration of fluorescently labeled E-box (black circles) and non-specific (open circles) sequences with 1:1 mixtures of c-Myc*/ΔMax*. (**B**) Populations of ΔMax*/Max* (blue), ΔMax*/c-Myc* (red) and ΔMax*/ΔMax* (pale blue) with the E-box (full lines) and the non-specific (dotted lines) sequences as a function of the total dimeric concentrations. (**C**) Titration of fluorescently labeled E-box (black circles) and non-specific (open circles) sequences with a 1:1 mixture of c-Myc*/Max. The lines were obtained from the simulation of the binding of 3 species: (**D**) c-Myc*/Max* (purple), Max*/Max* (blue) and c-Myc*/c-Myc* (red) for the E-box (full lines) and non-specific (dotted lines) sequences.

As depicted in Fig 7A, the titrations of the E-box and non-specific sequences with a 1:1 mixture of c-Myc* and ΔMax* leads to similar binding isotherm and fitted equilibrium constants (Table 1) indicating that the heterodimeric b-HLH-LZ can form similar amounts of complexes with both DNA sequences at 37°C. As shown in Fig 7B, the heterodimeric c-Myc*/ΔMax* can form equivalent and larger amounts of complexes with the E-box than ΔMax*/Max* and ΔMax*/ΔMax*, respectively. Interestingly, c-Myc*/ΔMax* make a larger population of complex with the non-specific sequence than both ΔMax*/Max* and ΔMax*/ΔMax*. This indicates that increasing the ratio of ΔMax over WT Max will lead to larger amounts of c-Myc to non-specific sequences and, albeit to a lesser extent, to E-box, increasing c-Myc tumorigenic activity.

**Fig 8:**
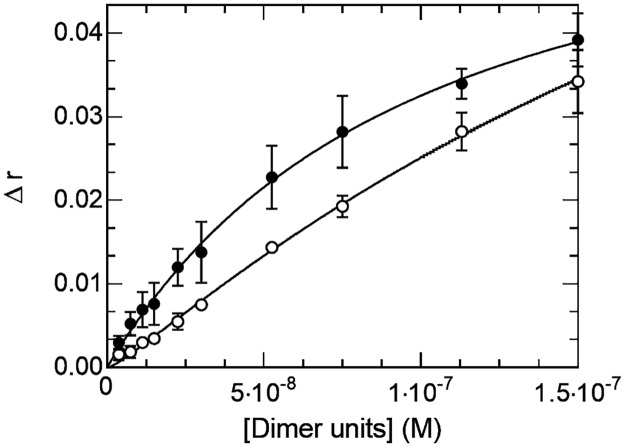
c-Myc* binds both specific and non-specific DNA sequences as a homodimer. Titration of fluorescently labeled E-box (black circles) and non-specific (open circles) sequences with c-Myc*.

For the sake of completeness, we present in Fig 7C the titration of the E-box and non-specific sequences with a 1:1 mixture of Max* and c-Myc*. Intriguingly, one can notice that the binding curves have atypical shapes. In fact, both curves could not be satisfactorily fitted to a single binding species (e.g. the heterodimer) suggesting that more than one complex is present as function of the concentration of c-Myc*/Max*. In accordance, we show on S3 Fig that even when c-Myc* is in a 2:1 excess, we can detect the Max*/Max* and c-Myc*/Max* DNA complexes migrating in EMSA showing- as described in detail elsewhere [23,26]- that the Max* homodimer can compete with the c-Myc*/Max* for DNA. As recently reported, we show by CD, fluorescence anisotropy and EMSA (S3 Fig) that c-Myc* can form a stable complex with the E-box [31]. In fact, whereas c-Myc* is often described as an unfolded and monomeric protein construct, it is certainly not completely a random coil and contains a significant amount of helical structure. Indeed, as described elsewhere [23,26] and shown in Fig 4, although the CD signal is rather weak, it contains less amount of random coil structure than ΔMax*, for instance. The notorious difficulties of working with c-Myc* reside in its poor solubility at or near neutral pH and at concentrations in the μM range. Accordingly, we show in Fig 8 that c-Myc* binds E-box and non-specific DNA sequences in the sub-micromolar concentration range, with significant apparent K_Binding_ and K_Dimer_. Hence, the concurrent accumulation of c-Myc*, as Max*/Max* binds to DNA, could lead to its binding to DNA. Although not statistically significant, we report on Fig 7 the results of a simulation of the binding of the three dimers to the E-box and non-specific sequence using the fitted parameters found in Table 1 for c-Myc*/c-Myc* and Max*/Max* and 1/K_Binding_ and 1/K_Dimer_ for c-Myc*/Max* smaller than those of Max*/Max*. As one can see, we were able to simulate the overall shapes of the binding curves with three populations of dimers binding to DNA, indicating that the c-Myc* homodimer can persist even when Max* is present. We refer to the studies of Hu et al. and Park et al. for alternative characterizations of the binding of the b-HLH-LZs of c-Myc and Max to DNA [27,29]. In these studies, the potential binding of different species has not been considered.

## Discussion

It was recently shown that high levels of c-Myc caused by the constitutive activity of EGFRvIII, a mutated form of EGFR, amplifies the transcription of hnRNPA1 in GBM and the alternative splicing of Max into its C-terminally truncated ΔMax isoform [11]. In fact, the ratio of ΔMax over WT Max is significantly increased in patients carrying the EGFRvIII mutation. Accordingly, the overexpression of ΔMax in GBM cell lines potentiates c-Myc mediated transcriptional transactivation of metabolic genes and cell proliferation. In contrast, overexpression of the WT Max significantly inhibits c-Myc transactivated transcription and markedly slows down proliferation. Although the lack of the C-terminal domain in ΔMax could play a direct role in the oncogenic role of ΔMax, the exact molecular explanation of such opposite roles between WT Max and this spliced isoform is still not known. Here, we focused on the biological effect of the change in primary structure in the LZ of ΔMax, also imparted by the splicing event, on its dimerization and DNA binding properties. We demonstrate that contrary to the WT motif (Max*), the b-HLH-LZ of ΔMax (ΔMax*) does not homodimerize in absence of DNA. Like Max*, ΔMax* can bind to the E-box sequence as a homodimer. However, contrary to its WT counterpart, ΔMax* cannot bind to non-specific DNA. Conversely, ΔMax* can heterodimerize with the b-HLH-LZ of c-Myc (c-Myc*) and bind the E-box and non-specific DNA with equivalent affinities. Similarly, ΔMax* can bind to E-box and non-specific DNA as a heterodimer with Max* but, in this heterodimeric form, leads to smaller populations of complex compared to the Max* homodimer and ΔMax*/c-Myc* heterodimer at equivalent dimeric concentrations. Such a behavior for the b-HLH-LZ of ΔMax is expected to directly impact on the amount of c-Myc bound to DNA and its transcriptional activity. Indeed, since ΔMax* does not bind non-specific DNA but preferentially heterodimerizes with c-Myc* and binds to the E-box sequence and non-specific DNA with comparable affinities, its overexpression in GBM cells can be anticipated to increase the amount of c-Myc at E-box sequences and non-specific sites, thereby increasing c-Myc transcriptional activities and induction of proliferation. In fact, in most cancer cells with elevated c-Myc, c-Myc/Max is found to accumulate at active enhancers and promoters, irrespective of the presence of E-box sequences, and activate the transcription of oncogenic programs [4,8,32]. This will be further promoted in a scenario where the ΔMax*/Max* heterodimer form less stable DNA complexes with both the E-box and non-specific sequences than Max*/Max*. Conversely, the homodimerization of Max and the ability of the homodimer to bind DNA (specific and non-specific) with high affinity limits the access of c-Myc/Max to DNA [9,23,26]. Hence, the overexpression of WT Max in GBM cells can be expected to reduce c-Myc transcriptional activities and proliferation, consistent with results reported by others [12–14,33].

Thus, for the first time, our data provide a mechanistic explanation for the fact that ΔMax has a pro-oncogenic function, whereas WT Max has the opposite effect. Additionally, this mechanism also offers a rationalization as to why a decrease in the WT Max/ΔMax ratio is associated with the aggressiveness of GBM tumors [11]. Indeed, the decrease in levels of WT Max in favor of ΔMax will limit the possibility of the Max homodimer to compete with Myc DNA binding as a heterodimer.

In summary, the splicing of WT Max into ΔMax is comparable to the inactivation of a tumor suppressor. In this regard, germline and somatic mutations in Max have been recently shown to be directly linked to hereditary and sporadic human pheochromocytoma, a cancer lesion affecting the adrenal gland [34–36]. More recently, inactivating mutations of Max in small cell lung cancer cell lines were also discovered to contribute to the tumorigenesis of this cancer type [37]. Somatic mutations in Max in other cancers are also reported in the COSMIC database. Many of those mutations are located at the dimerization interface and positions contacting DNA. Indeed, many of these residues mutated in pheochromocytomas are located in the basic region or the loop of Max (e.g. R25H, R35C and R60W). In a previous study, we have shown that the mutation of these residues in the complete gene product of Max led to a significant decrease in DNA binding affinity [38]. It was also recently shown that these mutant forms of Max were less effective than the WT at inhibiting Myc-dependent transcription of a reporter gene overexpressed in a pheochromocytoma cell line [34].

In conclusion, our results, combined to recent evidence from the literature, support the notion that the integrity of the primary structure of the b-HLH-LZ of Max and its ability to homodimerize and bind DNA with high affinity limits the genomic binding of c-Myc and its transcriptional activities. In a corollary fashion, modifications in the primary structure of Max that weaken its homodimerization and/or the DNA binding affinity of the homodimer, while preserving the ability to heterodimerize with Myc proteins, will have an oncogenic effect exacerbating genomic binding of c-Myc.

## Material and methods

### Cloning

The DNA coding for the b-HLH-LZ of Max (residue A13 to E94) and c-Myc (residue N353 to N436) were amplified by polymerase chain reaction (PCR) and inserted into pET3a plasmid (Novagen) as described elsewhere [23,25]. The sequence corresponding to the b-HLH-LZ of ΔMax was amplified and cloned into the pET3a plasmid (Novagen) using pET3a-Max* as template. The 5’-d(CCGCCATATGGCTGACAAACGGGCTCATCATAAT)-3’ and 5’-d(GGCGGGATCCCGACTCTGATTCGCCTTGCTGCTCCAGAAGAGCATTCA)-3’ oligos were used as 5’ and 3’ primers, which contain *Nde*I and *Bam*HI restriction sites, respectively. No stop codon was used. This resulted in the substitution of C-terminal amino acids VRALE to GESES with a C-terminal GSGC extension.

### Protein expression and purification

BL21 (DE3) pLysS competent *Escherichia coli* bacteria were transformed with the different Max and Myc b-HLH-LZ constructs and grown on LB agar supplemented with ampicillin (50 μg/mL) and chloramphenicol (34 μg/mL). For each mutant, one colony was selected and grown in 100 mL 2YT medium for 16 hours (pre-culture) and then diluted to 2% in a total of 5 L of 2YT medium containing ampicillin and chloramphenicol. When optical density (595 nm) reached 0.8, protein expression was induced with 0.5 mM IPTG and bacterial cultures were grown at 37°C overnight with agitation. Cells were harvested by centrifugation and lysed in high salt buffer. Protein extracts were centrifuged and proteins of interest were found in the inclusion bodies. The inclusion bodies were resolubilized in 6 M Urea, 0.5 M GuHCl, 100 mM NaAc buffer, pH 5.5. Proteins of interest were then purified on cation exchange chromatography columns (GE HiTrap SP-HP) using a salt gradient in citrate-phosphate buffer pH 2.8 after a wash with sodium acetate buffer pH 5.0. Highly pure fractions were desalted on HiTrap Desalting Columns (GE) using 0.05% H_2_O·TFA and lyophilized with 15% acetonitrile. Proteins were solubilized in PBS buffer pH 7.5 and their concentration were determined using optical density at 280nm and their respective molar extinction coefficients. Protein concentrations were verified by circular dichroism spectroscopy using established references.

### Circular Dichroism (CD)

A Jasco J-810 spectropolarimeter equipped with a Jasco Peltier-type thermostat was used to perform the CD measurements. The instrument was calibrated routinely with an aqueous solution of *d*-10-(+)-camphor-sulfonic acid at 290.5 nm. Quartz cells of 0.1 cm path length were filled with the samples containing protein buffered in 25 mM KH_2_PO_4_, 25 mM Na_2_HPO_4_, 50 mM KCl and 1 mM TCEP at pH 6.8. Spectra were recorded from 250 to 195 nm at 0.2 nm intervals by accumulating 5 scans at 20°C. Thermal denaturations were recorded at 222 nm from 5 to 95°C at a rate of 1°C/min. Single-stranded oligonucleotides were resuspended in Tris-EDTA buffer with 50 mM NaCl. Double stranded canonical E-box and non-specific DNA were obtained by heating the palindromic oligonucleotides [5’-GAG TAG CAC GTG CTA CTC-3’ and 5’-GAG TAG GGA TCC CTA CTC-3’ respectively] to 95°C and slowly cooling to room temperature in the heating block. The concentration of the dsDNA was determined by spectrophotometry at 260 nm. The DNA contribution to the CD signal was subtracted from the thermal denaturation curves containing DNA. Max* and ΔMax* were adjusted to a concentration of 12 μM, both in homo- and heterodimers, unless otherwise indicated. c-Myc* was used at a concentration of 8 μM both in homo- and heterodimers. Double-stranded canonical E-box and non-specific DNA were both used at a concentration of 16 μM.

### Fluorescence anisotropy

Fluorescence anisotropy measurements were performed at 37°C using a F-2500 Fluorescence Spectrophotometer (Hitachi) equipped with polarizers. Double stranded canonical E-box or non-specific DNA labeled with fluorescein [5’-GAG iFluorTAG CAC GTG CTA CTC-3’ and 5’-GAG iFluorTAG GGA TCC CTA CTC-3’ respectively] were obtained as described above. Solutions of dsDNA (15 nM) buffered in 2 mL of 50 mM Tris, 50 mM KCl, pH 7.5 were titrated using stock solutions of protein concentrated at 15 μM and pre-equilibrated 1h at 37°C. Each protein addition was followed by a 5-minute incubation at 37°C in order to reach equilibrium. Anisotropy values (r) were calculated using the following equation:

$$r=\frac{I_{∥}-I_{⊥}}{I_{∥}+2I_{⊥}}$$ (1)

where *I* is the fluorescence intensity parallel or perpendicular to the incident polarization. Optimal excitation and emission wavelength, 490 nm and 520 nm respectively, were determined using the fluorescence spectra of the labelled DNA. Slit widths were set to 10 nm. To monitor DNA binding, the increase (Δr) in anisotropy as a function of the concentration of protein in dimer units (Anisotropy([Dimer]) is reported and fitted with the following equation:

$$Anisotropy([Dimer])=P_{Complex}([Dimer])\cdot Anisotropy([Dimer]\to \infty )$$ (2)

where Anisotropy([Dimer]→∞) is the anisotropy measured at high concentrations of dimers and P_Complex_([Dimer]) is the actual population of complex at a given concentration define by the following equation:

$$P_{Complex}=\left(1+\left(\frac{\left[Dimer\right]}{\left[DNA\right]}\right)+\left(\frac{1/K_{Binding}}{\left[DNA\right]}\right)-\sqrt{{\left(1+\left(\frac{\left[Dimer\right]}{\left[DNA\right]}\right)+\left(\frac{1/K_{Binding}}{\left[DNA\right]}\right)\right)}^{2}-4\left(\frac{\left[Dimer\right]}{\left[DNA\right]}\right)}\right)/2$$ (3)

Because in absence of DNA the b-HLH-LZs are in equilibrium between their monomeric and dimeric species and that in the presence of DNA and at equilibrium the most stable bound species is the dimeric (homo- or hetero-) state, the population of complex will depend on the effective concentration of dimer [*Dimer*] = *P*_*Dimer*_ ∙ [*Dimer Units*], where *P*_*Dimer*_ is the effective population of dimer and [*Dimer Units*] is the total concentration of b-HLH-LZ ([*Dimer Units*] = [*Monomer Units*]/2). *P*_*Dimer*_ is obtained by *P*_*Dimer*_ = 1 − *P*_*Monomer*_, where *P*_*Monomer*_ is given by:

$$P_{Monomer}=\left(\frac{-\left(1/K_{Dimer}\right)-\sqrt{{\left(1/K_{Dimer}\right)}^{2}-16\cdot [DimerUnits]\cdot \left(1/K_{Dimer}\right)}}{8\cdot [DimerUnits]}\right)$$ (4)

where *1/K*_*Dimer*_ is the effective equilibrium dissociation constant (K_D_) expressed as the reciprocal of the equilibrium dimerization constant.

### Electrophoretic Mobility Shift Assay (EMSA)

Double stranded canonical E-box labeled with fluorescein [5’-GAG iFluorTAG CAC GTG CTA CTC-3’] was obtained as described above. Protein mixtures were pre-equilibrated for 30 minutes at room temperature. Double stranded DNA (250 nM) and proteins (166 to 1000 nM) were equilibrated together in binding buffer (20 mM Tris-HCl, 75 mM KCl, 2,5 mM TCEP, 25 μg/mL BSA, 5% Glycerol, pH 8.0) for 1h at room temperature. The resulting binding reactions were analyzed by migration on a 6% native polyacrylamide gel in TA buffer (40 mM Tris-acetate, pH 8.0) for 40 minutes at room temperature, 100V. The resulting image was obtained using a Molecular Imager VersaDoc^™^ MP 4000 System.

## Supporting information

S1 Fig χ^2^ of the fits. Obtained as a function of varying 1/K_Binding_ for the E-box (black circles) and non-specific (open circles) sequences for (**A**) Max*, (**B**) ΔMax*/Max*, (**C**) c-Myc*/ΔMax*, (**D**) c-Myc* and (**E**) ΔMax*. The 1/K_Dimer_ corresponding to the best fit is reported in Table 1.
(PDF)

S2 Fig Max* and ΔMax* binds to DNA as homo- and heterodimers. (**A**) Thermal denaturation of Max* in presence of E-box (black diamonds) and non-specific DNA (open diamonds) recorded by monitoring the CD signal at 222 nm. (**B**) Thermal denaturation of ΔMax* in presence of E-box (black triangles) and non-specific DNA (open triangles) recorded by monitoring the CD signal at 222 nm. (**C**) EMSA demonstrating that the migration of a fluorescently labeled E-box (250 nM) is retarded by the binding of c-Myc*, ΔMax*, ΔMax*/Max*, Max* and c-Myc*/Max* homodimeric and heterodimeric complexes. The protein concentration is indicated in nM. (**D**) Thermal denaturation of ΔMax*/E-box (open triangles), Max*/E-box (open diamonds) and ΔMax*/Max*/E-box (black diamonds) recorded by monitoring the CD signal at 222 nm and the arithmetic sum of the individual denaturations (gray diamonds). Note the presence of only one transition for the case of the mixture of ΔMax*/Max demonstrating that one (heterodimeric) complex is formed in the presence of DNA.
(PDF)

S3 Fig c-Myc* binds E-box sequences as a homodimer. (**A**) Thermal denaturation of c-Myc* in presence of E-box (black triangles) and non-specific DNA (open triangles) recorded by monitoring the CD signal at 222 nm. (**B**) EMSA demonstrating that the migration of a fluorescently labeled E-box (250 nM) is retarded by the binding of a mixture of Max*/Max* and c-Myc*/Max* when c- Myc* is in a 2:1 excess with Max*. The protein concentration is indicated in nM.
(PDF)

S1 Text Simulation details of the temperature denaturation curve of the c-Myc*/ΔMax* heterodimer. (PDF)
