# Supplementary material for: Biophysical characterization of the b-HLH-LZ of ΔMax, an alternatively spliced isoform of Max found in tumor cells: Towards the validation of a tumor suppressor role for the Max homodimers
Source: PLoS One. 2017 Mar 28;12(3):e0174413. doi: 10.1371/journal.pone.0174413 (PMC5370111; doi:10.1371/journal.pone.0174413)
Supplement: S1 Fig — Obtained as a function of varying 1/KBinding for the E-box (black circles) and non-specific (open circles) sequences for (A) Max*, (B) ΔMax*/Max*, (C) c-Myc*/ΔMax*, (D) c-Myc* and (E) ΔMax*. The 1/KDimer corresponding to the best fit is reported in Table 1. (PDF) [file pone.0174413.s001.pdf]

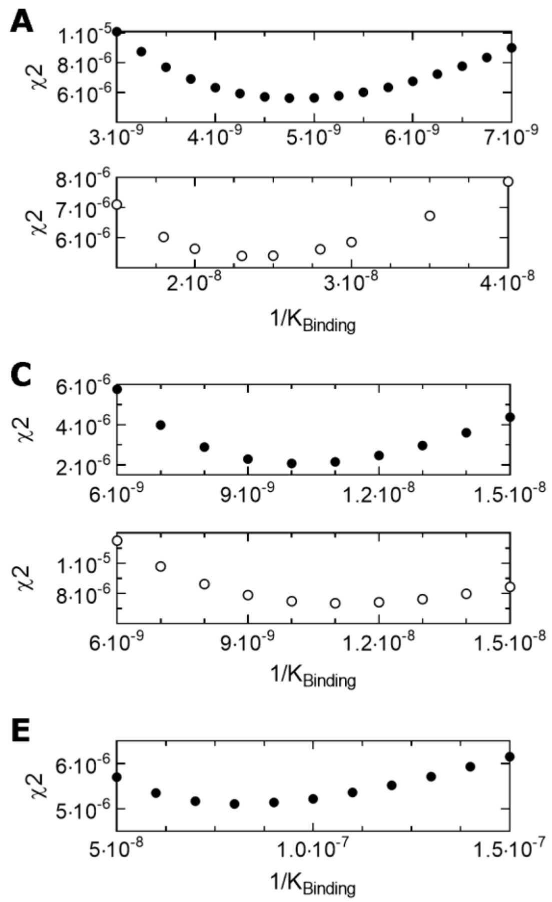

S1 Fig. χ^2 of the fits. Obtained as a function of varying $1/K_{Binding}$ for the E-box (black circles) and non-specific (open circles) sequences for (**A**) Max*, (**B**) ΔMax*/Max*, (**C**) c-Myc*/ΔMax*, (**D**) c-Myc* and (**E**) ΔMax*. The $1/K_{Dimer}$ corresponding to the best fit is reported in Table1.
