# Supplementary material for: Biophysical characterization of the b-HLH-LZ of ΔMax, an alternatively spliced isoform of Max found in tumor cells: Towards the validation of a tumor suppressor role for the Max homodimers
Source: PLoS One. 2017 Mar 28;12(3):e0174413. doi: 10.1371/journal.pone.0174413 (PMC5370111; doi:10.1371/journal.pone.0174413)
Supplement: S2 Fig — (A) Thermal denaturation of Max* in presence of E-box (black diamonds) and non-specific DNA (open diamonds) recorded by monitoring the CD signal at 222 nm. (B) Thermal denaturation of ΔMax* in presence of E-box (black triangles) and non-specific DNA (open triangles) recorded by monitoring the CD signal at 222 nm. (C) EMSA demonstrating that the migration of a fluorescently labeled E-box (250 nM) is retarded by the binding of c-Myc*, ΔMax*, ΔMax*/Max*, Max* and c-Myc*/Max* homodimeric and heterodimeric complexes. The protein concentration is indicated in nM. (D) Thermal denaturation of ΔMax*/E-box (open triangles), Max*/E-box (open diamonds) and ΔMax*/Max*/E-box (black diamonds) recorded by monitoring the CD signal at 222 nm and the arithmetic sum of the individual denaturations (gray diamonds). Note the presence of only one transition for the case of the mixture of ΔMax*/Max demonstrating that one (heterodimeric) complex is formed in the presence of DNA. (PDF) [file pone.0174413.s002.pdf]

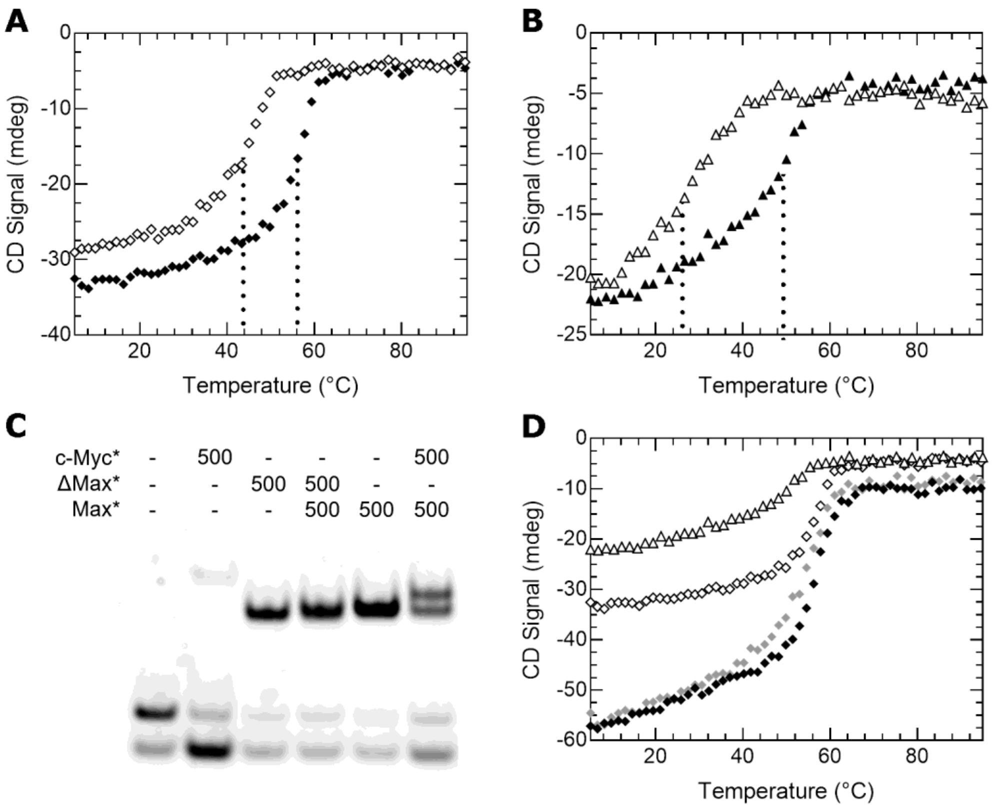

S2 Fig. Max* and ΔMax* binds to DNA as homo- and heterodimers. (**A**) Thermal denaturation of Max* in presence of E-box (black diamonds) and non-specific DNA (open diamonds) recorded by monitoring the CD signal at 222 nm. (**B**) Thermal denaturation of ΔMax* in presence of E-box (black triangles) and non-specific DNA (open triangles) recorded by monitoring the CD signal at 222 nm. (**C**) EMSA demonstrating that the migration of a fluorescently labeled E-box (250 nM) is retarded by the binding of c-Myc*, ΔMax*, ΔMax*/Max*, Max* and c-Myc*/Max* homodimeric and heterodimeric complexes. The protein concentration is indicated in nM. (**D**) Thermal denaturation of ΔMax*/E-box (open triangles), Max*/E-box (open diamonds) and ΔMax*/Max*/E-box (black diamonds) recorded by monitoring the CD signal at 222 nm and the arithmetic sum of the individual denaturations (gray diamonds). Note the presence of only one transition for the case of the mixture of ΔMax*/Max demonstrating that one (heterodimeric) complex is formed in the presence of DNA.
