# Supplementary material for: Biophysical characterization of the b-HLH-LZ of ΔMax, an alternatively spliced isoform of Max found in tumor cells: Towards the validation of a tumor suppressor role for the Max homodimers
Source: PLoS One. 2017 Mar 28;12(3):e0174413. doi: 10.1371/journal.pone.0174413 (PMC5370111; doi:10.1371/journal.pone.0174413)
Supplement: S3 Fig — (A) Thermal denaturation of c-Myc* in presence of E-box (black triangles) and non-specific DNA (open triangles) recorded by monitoring the CD signal at 222 nm. (B) EMSA demonstrating that the migration of a fluorescently labeled E-box (250 nM) is retarded by the binding of a mixture of Max*/Max* and c-Myc*/Max* when c- Myc* is in a 2:1 excess with Max*. The protein concentration is indicated in nM. (PDF) [file pone.0174413.s003.pdf]

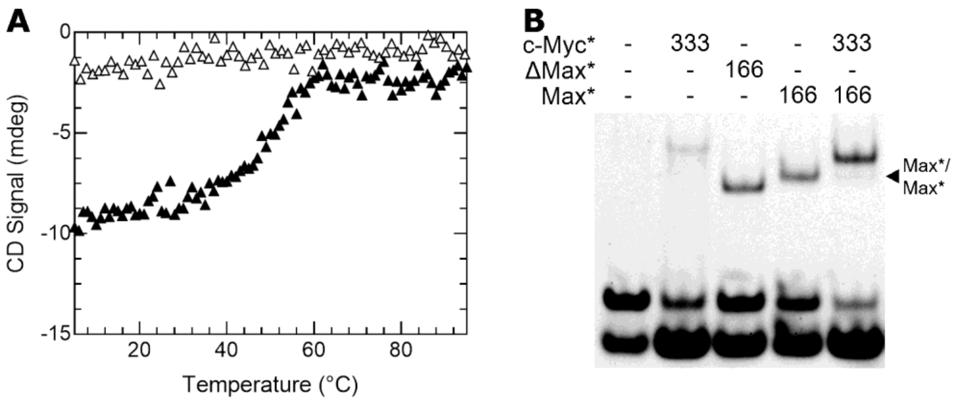

S3 Fig. c-Myc* binds E-box sequences as a homodimer. (**A**) Thermal denaturation of c-Myc* in presence of E-box (black triangles) and non-specific DNA (open triangles) recorded by monitoring the CD signal at 222 nm. (**B**) EMSA demonstrating that the migration of a fluorescently labeled E-box (250 nM) is retarded by the binding of a mixture of Max*/Max* and c-Myc*/Max* when c-Myc* is in a 2:1 excess with Max*. The protein concentration is indicated in nM.
