# Supplementary material for: Biophysical characterization of the b-HLH-LZ of ΔMax, an alternatively spliced isoform of Max found in tumor cells: Towards the validation of a tumor suppressor role for the Max homodimers
Source: PLoS One. 2017 Mar 28;12(3):e0174413. doi: 10.1371/journal.pone.0174413 (PMC5370111; doi:10.1371/journal.pone.0174413)
Supplement: S1 Text — (PDF) [file pone.0174413.s004.pdf]

S1 Text. Simulation details of the temperature denaturation curve of the c-Myc*/ΔMax* heterodimer.

The curve was simulated with the model of a dimer dissociating into two unfolded momomers as described in [23]. The enthalpy at T° (304.9 K ± 0.3 K), $\Delta H°_u(T°)$ was found to be 69.5 $kcal \cdot mol^{-1}$ ±3 $kcal \cdot mol^{-1}$. A $\Delta C°_{p,u}$ of 0.5 $kcal \cdot mol^{-1} \cdot K^{-1}$ was used in the procedure. $1/K_{Dimer}$ (20°C) and (37°C) values of and $4.9 \cdot 10^{-7}$ and $1.6 \cdot 10^{-4}$, respectively, were calculated from the stability curves ($\Delta G°_u(T)$) as described in [25].
